# MRI-Guided Radiation Therapy for Prostate Cancer: The Next Frontier in Ultrahypofractionation

**DOI:** 10.3390/cancers15184657

**Published:** 2023-09-21

**Authors:** Cecil M. Benitez, Michael L. Steinberg, Minsong Cao, X. Sharon Qi, James M. Lamb, Amar U. Kishan, Luca F. Valle

**Affiliations:** Department of Radiation Oncology, David Geffen School of Medicine, University of California Los Angeles, Los Angeles, CA 90095-6951, USAxqi@mednet.ucla.edu (X.S.Q.);

**Keywords:** prostate cancer, MRI, MRI-guided radiotherapy, radiotherapy, radiation oncology, SBRT, ultrahypofractionation

## Abstract

**Simple Summary:**

Prostate cancer treatment with radiation therapy has advanced significantly over the years. Leveraging the proposed low α/β ratio of prostate cancer, radiation therapy for the definitive treatment of upfront prostate cancer has moved from conventionally fractioned approaches to ultrahypofractionated fractionation schemes that require increased precision in both treatment planning and treatment delivery. MRI-guided radiation therapy (MRIgRT) enables this precision-based approach by improving visualization of the prostate and the surrounding structures, enabling safer dose escalation with reduced toxicity profiles relative to CT-guided radiation therapy. Real-time gating and on-table adaptive treatment further reduce dose to normal structures. Treatment efficacy and reduced toxicity must be balanced with ongoing challenges pertaining to MRIgRT workflow and training. In this review, we focus on the utility of MRIgRT in the treatment of prostate cancer and highlight ongoing trials using ultrafractionated MRIgRT.

**Abstract:**

Technological advances in MRI-guided radiation therapy (MRIgRT) have improved real-time visualization of the prostate and its surrounding structures over CT-guided radiation therapy. Seminal studies have demonstrated safe dose escalation achieved through ultrahypofractionation with MRIgRT due to planning target volume (PTV) margin reduction and treatment gating. On-table adaptation with MRI-based technologies can also incorporate real-time changes in target shape and volume and can reduce high doses of radiation to sensitive surrounding structures that may move into the treatment field. Ongoing clinical trials seek to refine ultrahypofractionated radiotherapy treatments for prostate cancer using MRIgRT. Though these studies have the potential to demonstrate improved biochemical control and reduced side effects, limitations concerning patient treatment times and operational workflows may preclude wide adoption of this technology outside of centers of excellence. In this review, we discuss the advantages and limitations of MRIgRT for prostate cancer, as well as clinical trials testing the efficacy and toxicity of ultrafractionation in patients with localized or post-prostatectomy recurrent prostate cancer.

## 1. Introduction

Prostate cancer is the second-leading cause of death among men with cancer, accounting for 34,500 male deaths per year in the United States [1]. Newly diagnosed prostate cancer is risk stratified based on clinical T staging, Gleason grade group, and serum PSA level [2]. Traditional upfront treatment approaches include radical prostatectomy (RP) and radiation therapy (RT) delivered as external beam radiation (EBRT) and/or brachytherapy. Depending on the risk stratification group of the patient, androgen deprivation therapy (ADT) is administered with radiation [2]. For locoregional recurrences post-prostatectomy, salvage RT can also be administered to the prostate bed [3].

Advances in the treatment of prostate cancer have made significant strides, partly due to technological advancements in radiotherapy delivery as well as a greater understanding of prostate cancer biology. Computerized tomography (CT)-based RT improved conformal treatment plans and made dose escalation possible. Seminal studies such as MRC RT01 showed superior biochemical control rates in patients treated with 74 Gy in 37 fractions when compared to patients that were treated with 64 Gy in 32 fractions [4]. RTOG 0126 demonstrated a 7-year biochemical control benefit of 70% with 79.2 Gy when compared to the 55% biochemical control benefit observed with doses of 70.2 Gy (*p* < 0.001) [5]. These trials established the benefit of higher doses of conventionally fractionated RT regimens.

Leveraging prostate cancer biology and its hypothesized low α/β ratio has galvanized clinical trials focusing on delivery of equally high biologically effective doses in fewer fractions while minimizing acute and late toxicities. Seminal papers such as CHHiP [6], RTOG-0415 [7], and PROFIT [8] investigated moderate hypofractionation. The UK CHHiP study demonstrated that 60 Gy in 20 fractions was noninferior to 74 Gy in 37 fractions, while RTOG 0415 showed that 70 Gy in 28 fractions was noninferior to 73.8 Gy in 41 fractions in patients with low-risk prostate cancer [6,7]. PROFIT established that 60 Gy in 20 fractions was noninferior to 78 Gy in 39 fractions in patients with low- to intermediate-risk prostate cancer not receiving androgen deprivation therapy (ADT) [8]. Results pertaining to early and late toxicity are mixed. RTOG 0415 showed a slight increase grade 3+ gastrointestinal (GI) toxicity of 4.6% in the moderately hypofractionated arm vs. 3% in the conventional arm, while grade 3+ genitorurinary (GU) toxicity was 4.5% in the hypofractionated arm and 6.4% in the conventional arm [7]. CHHiP and PROFIT did not identify significant differences in late gastrointestinal and genitourinary side effects [6,8]. Based on these studies and the patient convenience of completing RT courses over shorter time periods, moderate hypofractionation is now preferred over conventionally fractionated radiation therapy.

Additional studies have further increased dose per fraction using ultrahypofractionated approaches such as stereotactic body radiation therapy (SBRT), which is thought to better exploit the low α/β ratio in prostate cancer over conventional or moderate hypofractionation. HYPO-RT-PC demonstrated that 42.7 Gy in seven fractions was noninferior to conventionally fractionated prostate cancer in terms of failure-free survival in patients with intermediate- and high-risk prostate cancer. Though early side effects were more pronounced in the ultrahypofractionated group, late toxicity was similar in both regimens [9,10]. PACE B showed that in men with low-risk and favorable intermediate-risk prostate cancer, 36.25 Gy in 5 fractions over 2 weeks without ADT had similar toxicity rates at two years compared to conventionally fractionated RT at 78 Gy in 39 fractions or 62 Gy in 20 fractions over 4 weeks without ADT [11,12]. These studies demonstrated that ultrahypofractionated SBRT is feasible and has an acceptable safety profile when utilizing CT-based radiation therapy.

Because the oncologic outcome with SBRT is excellent and patients will live for many years after definitive treatment for localized prostate cancer, reducing long-term side effects is of outmost importance [13]. MRI-guided radiation therapy provides a potential avenue to reduce toxicity while maintaining high efficacy.

The development of the magnetic resonance imaging linear accelerator (MRI-Linac) has merged two seemingly technologically incompatible systems to improve visualization of soft-tissue structures and track target and organ-at-risk (OAR) positioning in real time. Currently, there are two FDA-approved commercial MRI-Linac machines that have been implemented and evaluated in clinical trials. The Unity MRI-Linac (Elekta, AB, Stockholm, Sweden) combines a 1.5 Tesla (i.e., high-field) MRI and a 7 MV FFF Elekta linear accelerator, while the MRIdian device (ViewRay Inc., Mountain View, CA, USA) combines a 6 MV linear accelerator with a 0.35 Tesla (i.e., low-field) MRI imaging system [14,15,16]. Both devices have been deployed in the evaluation of moderate or ultrahypofractionated treatment regimens for prostate cancer in the definitive and salvage settings. In this review, we will discuss the limitations and advantages of using MRIgRT to treat prostate cancer, as well as the ongoing clinical trials employing ultrahypofractionation using MRI-based platforms to usher in the next frontier of precision-guided prostate cancer treatments.

## 2. Limitations of CT-Based Treatment Planning and Delivery

CT-guided radiation therapy (CTgRT) is currently the most employed method for delivering radiation treatment for prostate cancer. However, the prostate, seminal vesicles, and surrounding OARs are known to move during and between fractions [17,18]. Specifically, bladder filling and rectal activity can displace the prostate, resulting in potential over- or underdosing of prostate targets. Nejad-Davarani et al. reported a 20.2% decrease in the dose to 95% of the PTV (D95%) due to prostate displacement over the course of 45 min [19]. Other studies have reported similar changes in prostate motion during the course of treatment, though minor effects with regard to prostate translation were observed when bladder volume changes were less than twofold [20].

To compensate in part for poor soft-tissue contrast visualization and interfraction and intrafraction movement, SBRT approaches require implantation of three radio-opaque fiducial markers for soft-tissue tracking [21]. The spatial distribution of the fiducial markers within the prostate must allow for 3D transformation. Fiducials that are too closely placed do not allow for accurate 3D prostate reconstruction [22]. Placement therefore requires technical expertise for optimal positioning. Fiducials can also migrate on average 1 mm between implantation, simulation, and treatment [23]. Though a migration of 1 mm may not be clinically significant with a large PTV margin, fiducial migration can limit the ability to reduce PTV margins with CT-based approaches.

Though fiducial markers serve as a surrogate for prostate position, they are unable to convey the shape of the prostate in real time. Rectal filling, bladder movement, and any other OAR volume changes are associated with prostate motion and shape [17,24,25]. ADT delivery is also known to alter prostate volume and molecular characteristics [26,27]. Changes in volume may also occur from radiation treatment itself over time, with increase in prostate size in the short term and a decrease in size in the long term. In patients that receive SBRT, the prostate volume can increase acutely by 21%. This increase is often most pronounced in the superior–inferior direction [28,29]. Hypofractionation with 20 fractions also results in an increase volume, though the increase has been reported to be more modest at 11% [28,30]. Unfortunately, there are no clinically implemented models that can predict anatomical changes and deformation with normal anatomical changes, nor are there protocols that evaluate how rectal and bladder filling might deform the prostate at the individual patient level in a predictable manner.

To account for uncertainty in prostate positioning and shape, the PTV margin is expanded from the clinical target volume (CTV). Typical PTV expansions for conventionally fractionated radiation is 5–9 mm isometrically, except posteriorly, where the expansion can often be decreased to 3–7 mm. In patients receiving SBRT, CTV to PTV expansion is typically 4–5 mm isometrically, except where the expansion can be decreased to 3–5 mm posteriorly [31,32]. The PTV margins regularly overlap with healthy bladder, rectum, and neurovascular structures. Dose-escalated SBRT treatment planning with CT-guided delivery must therefore proceeded with caution.

Dose-escalation studies using CT-guided SBRT have shown that there is a radiation dose limit. While incremental dose escalation from 32.5 Gy to 35 Gy to 37.5 Gy to 40 Gy improved biochemical failure rates without grade 3+ acute genitourinary toxicity and no late grade 3+ genitourinary and gastrointestinal toxicity [27], when doses were increased from 45 Gy to 47.5 Gy to 50 Gy there were significant increases in grade 4 gastrointestinal and genitourinary toxicities in spite of precautionary measures such as fleet enemas, rectal balloon placement, dexamethasone-use, and alpha-adrenergic antagonists [33]. These failed dose-escalation studies prompted caution for whole-gland dose escalation beyond 40 Gy using CT-guided radiation therapy and sparked interest in exploring other technological approaches for improving the therapeutic ratio.

## 3. Advantages of MRIgRT

**Figure 1:**
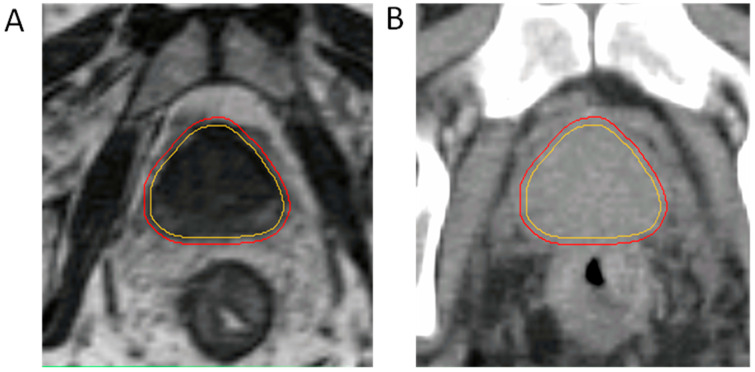
MRI-Linac-based contours on (**A**) the 0.35 Tesla MRIdian system and (**B**) a legacy CT planning scan. CTV is in yellow, isotropic 2 mm PTV is in red. Anatomic boundaries of the prostate are more clearly visualized with MRI guidance, enabling radical isotropic margin reduction from 4 mm to 2 mm. CT-only-based planning would result in a larger PTV margin than is currently displayed in image B.

MRIgRT has superior soft-tissue contrast over CTgRT and does not expose patients to additional ionizing radiation during image guidance or during treatment planning. Improved visualization of the prostate and OARs carries the potential to reduce gastrointestinal and genitourinary toxicity by reducing the PTV margins of uncertainty around the targets. The theoretical advantages to smaller PTV margins were recently shown to translate to important clinical benefits, including the ability to deliver modern dose-escalated SBRT while reducing both GI and GU toxicity. MIRAGE was a phase III single-center clinical trial from UCLA that randomized patients to receive 40 Gy in five fractions of SBRT using either an MRIgRT technique or a CTgRT technique. PTV margins were reduced to 2 mm isotropic margins for MRIgRT instead of the isotropic 4 mm margins used in CTgRT (Figure 1). This margin reduction decreased grade 2+ genitourinary toxicity by an absolute difference of 19% (24.4% vs. 43.4%, *p* = 0.01) and grade 2+ gastrointestinal toxicity by an absolute difference of 10.5% (0% vs. 10.4%, *p* = 0.003) [32]. Importantly, daily treatment adaptation was not performed in any of the patients enrolled on MIRAGE, and MR guidance alone was enough to reduce the toxicity seen with CTgRT. The additional benefit that daily treatment adaptation would have conferred in this setting remains under study.

Similar reductions in toxicity associated with MRIgRT have also been observed in the postoperative RT setting. In the multi-institutional phase II SCIMITAR study, men received 30–34 Gy in five fractions to the prostate bed using either CTgRT or MRIgRT depending on the technology that was available at the enrolling site. PTV expansion was reduced to 3 mm isotropically on MRIgRT and to 5 mm on CT-guided RT. In a post hoc evaluation of toxicity in men receiving MRIgRT vs. CTgRT, even in the absence of a defined prostate target, MRIgRT in the postoperative setting was associated with a 30.5% reduction in any grade acute GI toxicity [34]. The successor study to SCIMITAR is the actively accruing EXCALIBUR study (NCT4915508), which is designed similarly, albeit with all patients receiving MRIgRT, and will further determine early and late gastrointestinal and genitourinary toxicity associated with MRIgRT in the postoperative setting with a deformable target. Also in the postoperative setting, the SHORTER trial (NCT04422132) will randomize patients to either 36.5 Gy in 5 fractions or 55 Gy in 20 fractions using MRIgRT exclusively and will evaluate toxicity profiles between these moderately hypofractionated and ultrahypofractionated groups.

MRIgRT also presents as a potential treatment approach for reirradiation of prostate cancer after intraprostatic recurrence. Retrospective and preliminary studies have shown feasibility and tolerable toxicity in patients receiving total doses ranging from 25–40 Gy in five fractions. In such studies, no grade 2+ GU toxicity was observed, while grade 2+ GI toxicity occurred in 4 out of 18 patients (22.2%) [35,36]. Two patients were adopted with good tolerance but without substantial differences in GI toxicity compared to patients without online adaptation.

**Figure 2:**
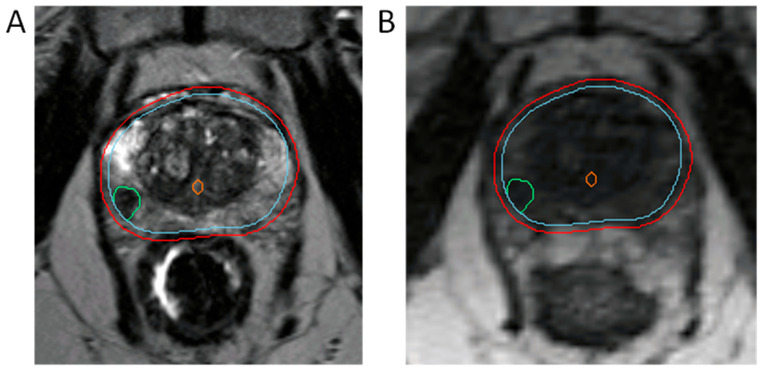
MR-Linac-based integration of simultaneous integrated boosting of the dominant intraprostatic lesion (DIL). (**A**) T2-weighted diagnostic MRI. (**B**) MR-based planning image on the MRIdian device. DIL is in green, urethra is in orange, CTV is in cyan, 2 mm isotropic PTV expansion is in red.

Because toxicity increases with whole-gland SBRT doses above 40 Gy, clinical trials have focused on boosting the dominant intraprostatic lesion (DIL) instead, given that this is among the most common sites of local radiorecurrence [37]. Specifically in the Unity, Elekta systems, DILs can be easily visualized and contoured directly on the simulation images. In contrast, rigid registration of MR simulation images to diagnostic multiparametric MRI images is adopted for DIL identification using the MRIdian system (Figure 2). The benefit of simultaneous integrated boosting of DILs was initially demonstrated in the FLAME trial, where a simultaneous integrated boost (SIB) to the DIL(s) in patients receiving conventional radiation improved biochemical disease-free survival with no significant differences in toxicity and quality of life in patients with intermediate- and high-risk prostate cancer [38]. Of note, OAR constraints were rigidly prioritized over boost target coverage in order to achieve isotoxicity. Focal lesion boosting in patients receiving moderately hypofractionated radiation, as explored in the DELINEATE trial [31], was similarly shown to be safe and effective. There is also interest in applying this technique to the ultrahypofractionated setting, with the hypo-FLAME trial seeking to simultaneously boost DILs to 50 Gy in five fractions while the remaining whole prostate gland is de-escalated to receive 35 Gy in five fractions. Results thus far demonstrate favorable rates of grade 2 GI and GU toxicity [39]. The long-term benefit of SIB to ultrahypofractionated RT is eagerly awaited. Regardless, MRIgRT offers an ideal platform for accurately delivering SIBs to DILs.

Improved anatomic visualization of OARs with MRIgRT may also assist in reducing doses to the urethra and surrounding vascular structures responsible for erectile function, and thus has the potential of minimizing toxicity in these critical domains. Late radiation toxicity to the urethra can cause urethral stricture formation. Using MRI-guided adaptive radiotherapy, patients treated with 36.25 Gy in five fractions with urethral doses up to 6.5 Gy per fraction had favorable GI and GU toxicity compared to historical controls and no grade 3+ GU toxicity [40,41], likely owing to confident visualization of the urethra thereby limiting dosimetric hot spots in this OAR. Techniques to improve visualization of the urethra on low-field MRIgRT devices are also under active study [42,43,44]. Similarly, radiation to the vascular structures contributes to the development of erectile dysfunction. Sparing vascular structures such as the pudendal artery in patients receiving conventional fractionation for localized prostate cancer was shown to preserve erectile function when compared to historical controls [45]. The ERECT trial (NCT 04861194) will evaluate erectile function over three years after localized MRI-guided SBRT to the prostate with sparing of the neurovascular bundles, internal pudendal arteries, corpora cavernosa, and penile bulb [46].

**Figure 3:**
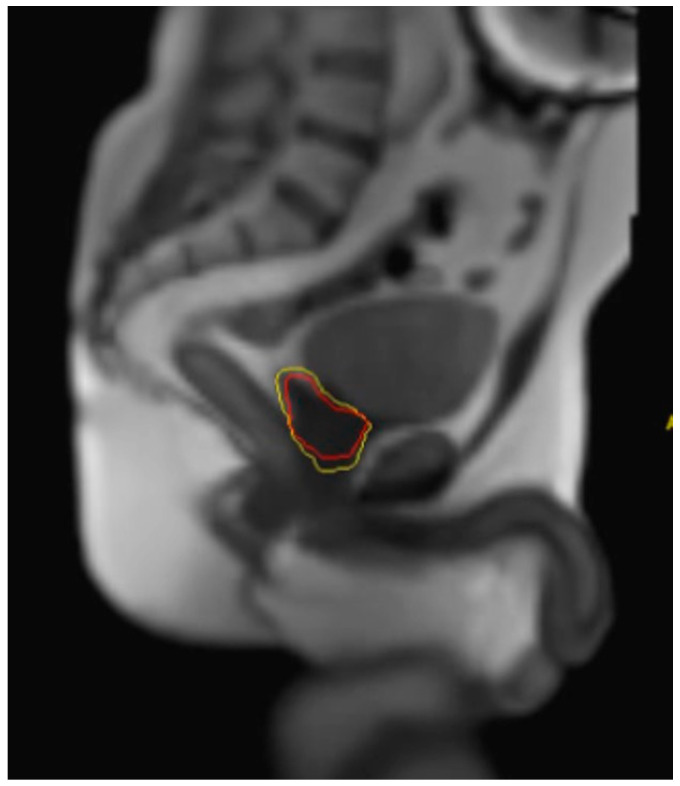
MR-Linac-based interface on the MRIdian device showing the prespecified 3 mm gating margin (yellow) and prostate CTV (red). These images are acquired in the sagittal plain at 4 frames per second during treatment delivery.

Another advantage to MRIgRT is the option for direct soft-tissue-based gating. Unlike legacy CT-based gating strategies, MRIgRT allows for real-time imaging of the prostate CTV through the acquisition of orthogonal imaging planes at a rate of four to eight frames per second while the RT is underway. Gating thus allows the operator to set parameters that will automatically hold the beam should there be substantial motion outside of the predefined gating parameters. An example of parameters in active clinical use at UCLA includes an allowable threshold of 10% of the prostate CTV outside of a 3 mm gating margin (Figure 3). Each gating parameter may be institution-specific based on physician preferences and institutional workflows, though strict gating parameters must be balanced with prolonged treatment times.

MRIgRT also has the potential advantage of on-table adaptation, which, though not strictly necessary to improve radiation toxicity per the MIRAGE trial [32], does have the potential to reduce toxicity even further in patients treated for localized prostate cancer [41] and locally recurrent prostate cancer [47]. This is especially useful if OARs such as the rectum move within the radiation field or if treatment targets such as the seminal vesicles are displaced such that they overlap with rectal structures. Online adaptive treatment may have even more value in the post-prostatectomy setting, as Nikitas et al. found that 48.4% (75/155) of fractions failed at least one OAR dose constraint [47], whereas when following adaptive planning, 77.4% (120/155) of fractions met OAR constraints. An adaptive workflow allows for real-time replanning and treatment delivery, thus minimizing the impact of deleterious anatomic changes between fractions.

The adaptive workflow is available in both Unity and ViewRay systems. Specifically, the Elekta Unity MRI-Linac has two online planning methods: adapt-to-position (ATP) and adapt-to-shape (ATS), both of which require a reference image from initial planning [14]. The reference plan is generated using the Monaco treatment planning system (TPS) using the GPUMCD dose calculation algorithm, and secondary dose calculations are generated with the Raystation TPS [48]. In the ATP workflow, the reference image is registered to the daily MR image and it is adapted to the translations using only rigid registration. This method does not account for changes in shape or OARs [49], and addresses Unity’s limitations of couch movement laterally and vertically. The ATS workflow instead takes the reference image and deformably registers it to the daily MRI. Contours are then propagated either rigidly or deformably. The physician is able to edit both target and OAR volumes based on the daily anatomy seen on the session MRI. Then, an online plan is generated, and quality assurance is carried out to ensure that the plan parameters are met. The MRIdian workflow also allows for recontouring of target and OAR structures [50]. Doses to the new OARs are predicted and then plan reoptimization is used to improve dosimetry. The reoptimized plan is verified and approved with an online quality assurance step as well. A repeat MRI can be taken if deemed necessary to verify positional accuracy prior to treatment delivery.

## 4. Limitations of MRIgRT

As patients susceptible to prostate cancer continue to be diagnosed at late stages of life, the likelihood of these men having comorbid illnesses that result in the placement of implantable cardiac devices and/or metal prosthesis is not remote. These devices may preclude the use of MRI-based techniques in certain situations. Metal prostheses and vascular stents, especially if located within the pelvis, can lead to geometric distortions and signal loss in surrounding structures, which can interfere with target delineation and daily setup efforts as well as dose calculations. A pilot study used B0 mapping at the time of simulation to estimate the expected distortions in images acquired during MRI simulations in patients with hip prosthesis [51]. These data were used to determine if the expected distortions during MRIgRT would be acceptable. If acceptable, a subset of these patients with prosthesis could be treated with MRIgRT. B0 mapping, however, is not commercially available on the current MRI-Linac platforms. Thus, to account for uncertainties associated with distortions, a larger PTV margin may be required.

Other devices that complicate MRIgRT include pacemakers. Though not an absolute contraindication, in patients that are pacemaker-dependent, additional measures must be taken that involve collaboration with electrophysiologists, device representatives, and radiation therapists at multiple points prior to MR simulation, during treatment planning, and delivery. Institutional-based workflows have been established and demonstrated for both 1.5 T and 0.35 T systems [52,53].

Additionally, MRI-Linac devices resemble traditional MRIs with a closed bore that may make it difficult to treat patients with a larger body habitus or patients with severe claustrophobia and anxiety. Further, the craniocaudal field length for the Unity device is 22 cm, and it is 24 cm for the MRIdian device [54]. This field length is sufficient for patients that receive definitive treatment to the prostate and prostate bed; however, treatment of larger fields, including the pelvic lymph nodes, may prove to be more challenging in specific cases. Though elective irradiation to the pelvic nodes is controversial, data do support elective nodal radiation in patients with high-risk disease [54] as well as for patients treated in the post-prostatectomy setting per RTOG 0534 [55]. Inclusion of elective pelvic nodes and/or nodal boosting in patients with gross pelvic nodal disease may thus not be uniformly suitable for treatment on MRI-Linacs, especially when treating on the Elekta Unity device.

Though soft-tissue contrast resolution is improved with MRIgRT compared to CTgRT, there are resolution differences between the Elekta Unity device and MRIdian. The lower 0.35 Tesla magnet strength on the MRIdian device limits direct visualization of dominant intraprostatic lesions during treatment planning, necessitating registration of the diagnostic MRI which has the potential to introduce contouring errors. Further, neoadjuvant initiation of ADT may make it difficult to identity DILs on both devices.

MRIgRT delivery is also more complex and requires longer treatment times compared to CT-guided radiation. Typically, on-table time with CT-guided SBRT treatments ranges from 15–30 min, whereas treatment times can be prolonged to 30–45 min with MRIgRT systems. If adaptive radiotherapy is employed, the on-treatment time may be extended even further to 45–60 min [49,56,57]. Longer treatment times may be more difficult to tolerate for patients instructed to maintain a full bladder during treatment to physically displace the small bowel out of the treatment field.

Moreover, treatment expediency must be balanced with treatment accuracy. Gating margins as well as the allowable threshold of CTV outside of the gating margin will also influence on-table treatment times. Strict gating boundaries increase the likelihood that the prostate will be outside the allowable margin at the expense of extended treatment times. However, despite longer treatment times, patients have reported high satisfaction when undergoing MRIgRT [56]. While some studies have shown that beam gating may only have small dosimetric impacts on online-adaptive MRIgRT, these studies did, in fact, implement “manual gating”, where dose delivery was interrupted in the majority of patients to initiate 3D couch corrections after large displacements noticed on the onboard MRI [58].

Novel treatment platforms such as MRIgRT also require increased training for clinicians and therapists, and, thus, can present workflow challenges. Increases in downtime with this technology can further impact the efficiency of treatment. Workflow paradigms are being rigorously developed to improve efficiency while maintaining strict quality and assurance measures.

## 5. Future Directions

Wide adoption of MRIgRT for the treatment of prostate cancer necessitates improvements in the workflow from initial planning to treatment delivery. Because MRI has no direct relationship between signal intensity and electron density, CT imaging is often obtained in addition to the MRI simulation images for treatment planning and patient positioning. These images are then coregistered during the treatment planning process, which has the potential to introduce uncertainties in dose calculation. Development of MRI-only radiation therapy without the need for CT-based imaging is underway [59]. Three main approaches to develop synthetic CT imaging include bulk density override, atlas-based, and voxel-based techniques. Bulk density override assigns an electron density to the entire patient volume. This technique assumes homogenous density across the volume and can result in a 2% error in MRI dose calculation [60,61,62]. The atlas-based technique uses a single MRI sequence to produce a synthetic CT [63]. Voxel-based techniques include the acquisition of both routine and specialized MRI sequences, namely, ultrashort echo time imaging, which can prolong patient treatment times [64,65]. Studies using atlas-based and voxel-based techniques have reported a dosimetric difference of <1% between the synthetic CT and planning CT with <1 mm deviations when synthetic CTs are used for positional verification [59]. Optimization of MRI-only planning will not only improve workflow but also reduce additional exposure to ionization radiation for patients.

The need for full bladder preparation is also under evaluation. Since treatments using MRI-Linacs take longer than CT-based machines, multiple studies have evaluated if changes in bladder filling can still meet bladder constraints consistently. For patients treated on the Unity system, Smith et al. found that all mandatory bladder constraints and 99.1% of bowel constraints were achieved at the time of treatment with a wide range of bladder volumes (175 mL to 525 mL) [66]. These results suggests that strict adherence to bladder-filling protocols may not be necessary to meet dose constraints with MRIgRT. Another approach is to use adaptive radiotherapy to compensate for interfraction change in bladder positioning and filling [40,41]. Simplifying bladder preparation may also reduce stress associated with achieving a full bladder.

On-table adaptation further increases treatment times because this requires the clinician or dosimetry team to recontour the target and the surrounding structures [67]. Some studies have focused on training nonclinician radiographers to contour. Preliminary studies show that clinician-generated volumes and radiographer-generated volumes resulted in comparable and clinically acceptable plans [68]. Alternatively, automating contours during on-table adaptive radiotherapy can also improve workflow efficiency. Accordingly, the use of artificial-intelligence-based contouring platforms to delineate the prostate, seminal vesicles, rectum, anal canal, bladder, penile bulb, and bony structures resulted in 80% of contours rated as clinical acceptable, 16% requiring minor adjustments, and 4% needing major adjustments [69,70].

In parallel, clinical trials are evaluating the efficacy and toxicity profiles of various ultrahypofractionation schemes that further reduce the total number of fractions delivered while increasing the dose per fraction. For example, the FORT study (NCT 04984343) is a noninferiority randomized trial on the MRIdian device that will compare 25 Gy in two fractions delivered to the prostate to 37.5 Gy in five fractions. The HERMES trial (NCT 04595019) will compare 36.25 Gy in five fractions delivered to the prostate vs. 24 Gy in two fractions delivered to the prostate with an intraprostatic boost to 27 Gy in two fractions. This study will evaluate acute grade 2+ genitourinary toxicity as the primary outcome. iSMART (NCT 05600400) will also compare 27 Gy in two fractions to the more established regimen of 40 Gy in five fractions.

Other approaches aimed at including efficacy with MRIgRT platforms include simultaneous boosting of the DILs, which tend to be the most common site of local recurrence after EBRT. AFFIRM (NCT 05373316) will evaluate gastrointestinal and genitourinary toxicity associated with patients treated with 35 Gy to the prostate and a boost to 50 Gy in five fractions. Boost-MSK (NCT 04997018) will access the reduction in post-treatment biopsy rates at 24 months in patients treated with 40 Gy in five fractions to the prostate and 45 Gy to the dominant lesion using the Unity platform. SIBRT (NCT 03664193) will evaluate the feasibility of delivering 35 Gy in five fractions to the prostate with an SIB to 37.5 Gy, 40 Gy, 42.5 Gy, or 45 Gy. Other approaches, such as in LEAD (NCT 0411319), include using the MRIdian platform to deliver 12–14 Gy in one fraction to the multiparametric MRI-defined GTV on day 1 followed by standard fractionation over 38 sessions with IMRT.

From an economic perspective, multiple studies have evaluated if MRIgRT is cost-effective when compared to 5, 20, and 39 fraction schedules of EBRT. Cost-effectiveness comparisons must be weighed against the extent of side-effect reduction, biochemical control, and mortality. Some studies have proposed that hypofractionated schedules using MRIgRT are reasonably expected to be cost-effective [71], while other studies have shown that if MRI-Linacs are to be cost-effective, grade 2+ urinary and gastrointestinal complications must be reduced by approximately 50% [72]. Studies such as MIRAGE have demonstrated improvements that meet this bar, though additional improvements in workflow (i.e., through implementation of MRI-only radiotherapy) can further reduce costs [32,73]. MRI-Linac approaches must also be weighed against alternative approaches such as ultrasound-guided radiation therapy [74,75].

## 6. Conclusions

MRIgRT offers numerous advantages (both theoretical and actual) over CT-based radiation therapy platforms, including improved soft-tissue contrast resulting in superior target and OAR visibility, the ability to deploy real-time motion tracking, and potential for on-table adaptive treatment. Phase III randomized data in patients treated for localized prostate cancer with MRIgRT have demonstrated improved toxicity profiles associated with PTV margin reduction. Additional ongoing studies are evaluating the benefit of MRIgRT in the post-prostatectomy setting. Current studies are focusing on improving workflow procedures through the use of synthetic CT MRI-only radiation, autocontouring during on-table adaptation, and further reduction in fractionation. Shortened treatment times must be balanced with quality and safety, while evaluation of cost-effectiveness will need to incorporate advances in workflow efficiency, locoregional control, and early and late toxicity.
